# Gendered socioeconomic impacts of emerging infectious diseases: Insights from a mixed-methods study in Guinea^☆^

**DOI:** 10.1016/j.onehlt.2026.101525

**Published:** 2026-07-19

**Authors:** Stéphanie Maltais, Janyck Beaulieu, Castro Gbêmêmali Hounmenou, Salifou Talassone Bangoura, Gnouma Laurent Koniono, Emile Faya Bongono, Alioune Camara, Justin Masumu, Abdoulaye Touré

**Affiliations:** a Department of Health Management, Evaluation and Policy, School of Public Health, Université de Montréal, Canada; b Centre for Public Health Research, Université de Montréal and CIUSSS du Centre-Sud-de-l'Île-de-Montréal, Canada; c Research Group on Epidemiology of Zoonoses and Public Health (GREZOSP), Faculty of Veterinary Medicine, Université de Montréal, Canada; d School of International Development and Global Studies, University of Ottawa, Canada; e Centre de Recherche et de Formation en Infectiologie de Guinée (CERFIG), Gamal Abdel Nasser University, Conakry, Guinea; f Computer Science Department, University of Labe, Guinea; g Public Health Department, Faculty of Sciences and Health Techniques, Gamal Abdel Nasser University, Conakry, Guinea; h Département de Médecine Vétérinaire de l'Institut Supérieur des Sciences et de Médecine Vétérinaire de Dalaba, Guinea; i Institut national pour la recherche biomédicale (INRB), Democratic Republic of the Congo

**Keywords:** Gender, Intersectionality, Socioeconomic impacts, Infectious disease outbreaks, One health, Guinea

## Abstract

This study examines the gendered socioeconomic impacts of infectious disease outbreaks, including both epidemics (e.g., yellow fever, Ebola, Marburg and Mpox) and pandemics (e.g., COVID-19), on populations in Guinea, using data from the *Decentralize and Operationalize One Health Platforms* (DOPERAUS) project. A mixed-methods approach combined quantitative survey data (*N* = 906) from participants aged 18 and over and qualitative data from an open-ended questionnaire answered by 508 respondents to capture both measurable outcomes and nuanced experiences. Sampling accounted for gender, age, and vulnerable populations. Analyses incorporated descriptive statistics, Chi-square tests, Wilcoxon-Mann-Whitney tests, and thematic coding of qualitative data.

Findings reveal gendered disparities in how socioeconomic impacts are experienced during infectious disease outbreaks. Women disproportionately bore caregiving and household management responsibilities and were more affected by income loss and reduced market access, as many reported being unable to sell their goods in the qualitative data. Men were more affected by income loss and disruptions to traditional gender roles based on the quantitative data. Vulnerability was also shaped by age and occupational status, with adults aged 30–49 and those in informal or precarious employment experiencing heightened economic and social stress. Women frequently relied on informal borrowing and community support to cope with financial strain, highlighting structural gaps in social protection systems.

These results underscore the intersectional nature of infectious disease outbreaks impacts, showing that gender alone does not fully explain vulnerability. The study emphasizes the importance of integrating social science perspectives into the One Health approach. Understanding household dynamics, community roles, and economic inequalities is essential for designing effective and equitable outbreak preparedness and response strategies.

## Highlights

• In Guinea, women face higher caregiving and economic precarity during infectious disease outbreaks.
• Women in informal work faced more financial stress, relying on community support.
• Integrating social sciences into the One Health approach helps capture household and economic dynamics.
• Gender-sensitive policies and strong community safety nets improve infectious disease outbreaks resilience.

## 1 Introduction

One of the most striking features of the COVID-19 pandemic, and other epidemic-prone zoonotic diseases such as Marburg virus disease, anthrax, influenza, mpox, chikungunya, and Ebola, is their unequal distribution across and within countries. Infectious disease outbreaks tend to disproportionately affect certain communities and individuals, increasing their vulnerability to diseases with epidemic potential and their associated socioeconomic consequences [1]. In that regard, a growing body of literature has highlighted a range of factors that influence the incidence and outcomes of COVID-19 cases globally, including socioeconomic status, gender, age, ethnicity, and political context [1], [2], [3], [4], [5], [6].

Various social determinants influence human-animal interactions, potentially impacting the prevalence of zoonotic diseases in specific areas. These determinants can be grouped into seven categories including: biological characteristics (e.g., age, sex, immune status, comorbidities); social characteristics (e.g., gender, family structure, food security, location, poverty, ethnicity, class, and race); norms (e.g., animals in the house, food preferences, hygiene and sanitation); livelihood systems (e.g., agricultural practices, hunting, gathering); settlement patterns (e.g., housing construction, density of housing, distance from forests); local and national governance and politics; and international governance and politics which include policy, regulations and enforcement [7]. Recognizing that specific communities and populations bear a greater burden of diseases with epidemic potential, especially zoonotic diseases, underscores the importance of comprehending these contexts to both prevent and effectively address epidemics.

Gender is primarily understood as a process through which individuals learn to differentiate between established and socially constructed characteristics associated with femininity and masculinity [8]. Social and domestic roles shaped by gender can influence an individual's likelihood of exposure to hazards, including infectious diseases and zoonoses [9]. Zika and the Ebola virus epidemics have underscored a lack of attention to gender-specific needs during the response to these health emergencies, highlighting a gender-blind approach [10]. The impacts of disease outbreaks are shaped by both biological factors and socially constructed gender roles. The accessibility of healthcare services, engagement with emergency personnel, and the design of health system governance all reflect underlying gender-based disparities [8].

Intersectionality is a feminist theoretical framework originally coined by Kimberlé Crenshaw that acknowledges how individuals can experience multiple layers of social identity simultaneously, in relation to discrimination, inequality and oppression, such as gender, race, class, and sexual orientation. [11]. These intersecting identities can create unique experiences and vulnerabilities. For example, a woman from a marginalized racial group may face different health challenges compared to a white woman of a similar socioeconomic status. By employing an intersectional perspective, we can shed light on and analyze the intricacies of how various forms of oppression intersect within individuals, impacting their mental and physical well-being [12]. This approach yields fresh insights and more comprehensive portrayals of the experiences of marginalized groups and the underlying factors that shape these experiences. Consequently, it fosters enhanced comprehension of health issues and the development of more holistic solutions [13].

The link between gender, intersectionality and the One Health (OH) approach is evident in the recognition that health challenges are influenced by multiple factors, including gender, socioeconomic status, race/ethnicity, environmental conditions and climate change [10], [11], [14], [15]. When applying the OH approach, it is essential to consider the diverse backgrounds and experiences of individuals and communities. For example, a OH intervention for a zoonotic disease may need to account for how gender roles and access to healthcare affect the vulnerability of different groups within a community [16].

Table 1 summarizes the key health and socioeconomic impacts of health crises reported in the literature, highlighting the ways in which gender shapes these impacts across different domains.

**Table 1:** Gendered health and socioeconomic impacts of (re)emerging infectious diseases on populations.

Type of Impacts	Specific Effects and Manifestations	Gender Dimensions
Health Impacts	• Direct morbidity and mortality especially among women [17] • Access to care limitations [17] • Mental health consequences (anxiety, grief, trauma, etc.) [8], [18], [19], [20], [21] • Long-term illness (e.g., long COVID) [22], [23] • Inadequate support for home-base care and absence of home-based care providers [8]	• Women delay care due to caregiving roles [17], [18] • Overrepresentation of women in frontline, and women of colour are especially likely to be employed in healthcare position in direct contact with patients [8], [15], [17] • Restricted access to sexual and reproductive services [15], [19] • Increased number of adolescent pregnancies [19]
Socioeconomic Impacts	• Loss of income or employment [19] • Food insecurity; reduced ability to purchase nutritious foods; reduced household purchasing power [19], [24] • Interruptions to markets, trade and supply chain; food shortages; rise of price levels due to disruption in supply chain [19], [24]	• Underpaid and informal roles of women in response to outbreaks [8], [15] • Informal jobs held by women lost first [15], [18], [19] • Food insecurity especially among female-headed households or informal workers [15]. • Gendered economic fallout (e.g. women-owned businesses disrupted) [15]

While we recognize the broader sociocultural impacts of health crises on affected populations, these dimensions will be examined in a separate publication.

## 2 Material and methods

The study employed a mixed-methods design. A survey was conducted in Guinea in 2023 to explore how infectious disease outbreaks, in this case mostly Ebola and COVID-19, affect woman and other marginalized populations.

### 2.1 Study population and sampling

The study population included individuals aged 18 years or older who had lived in one of the selected study sites for at least three months and had voluntarily consented to participate. We used a two-stage random sampling strategy, first selecting households from regional capitals using 2014 census data, then randomly choosing two to three adults from each household for participation.

The planned sample included 422 households distributed proportionally across all administrative regions based on their population weight. Data were collected in eight sites with varying numbers of households surveyed: Boké (*N* = 55), Conakry (*N* = 82), Faranah (*N* = 31), Kankan (*N* = 42), Kindia (*N* = 56), Labé (*N* = 51), Mamou (*N* = 52), and N'zérékoré (*N* = 53). Data teams received household lists with contact information, verified households three days prior, and administered questionnaires to two or three randomly selected adults per household, ensuring inclusion of women, older adults, people with disabilities, and pregnant women.

The sample included 906 individuals, predominantly female (71,6%), with a median age of 30 years. The qualitative component included responses from 508 participants who completed the open-ended questions, providing detailed accounts of their experiences. Although more women than men participated, this was not intentional; it reflects the composition of available respondents in the selected households rather than a deliberate sampling bias.

### 2.2 Data analysis

#### 2.2.1 Qualitative analysis

Responses to qualitative questions were recorded with participants' consent, fully transcribed, and translated by research assistants. A coding framework was developed using a combined deductive-inductive approach, based on the study objectives, relevant literature, and emergent themes from the data. Thematic analysis was conducted in NVivo following Braun and Clarke's six-phase framework [25]. To enhance credibility and reduce potential bias, two researchers independently coded the data. Key themes identified included gender roles, access to care, sociocultural norms, mental health, stigmatization, food insecurity, poverty, precarity and other gendered experiences.

#### 2.2.2 Quantitative analysis

Quantitative data were prepared for descriptive analyses, with missing age values imputed using the median. Variables were coded by measurement level (categorical: gender, education, occupation; ordinal: policy evaluations) and analyzed in R 4.2.2 (tidyverse, stats). Descriptive statistics included medians and IQRs for age, frequency distributions for categorical variables, Chi-square tests, and Wilcoxon-Mann-Whitney tests for nonparametric age comparisons.

### 2.3 Ethical considerations

The Guinean National Health Research Ethics Committee approved this study (authorization no. 025/CNERS/23). Ethical approval was granted by the University of Ottawa (S-10-22-8549), where one of the authors was formerly affiliated, and this approval was later acknowledged by the Research Ethics Committee in Science and Health (CERSES) at the Université de Montréal (Project 2026–8316) to align with her current institutional affiliation. Written, free, and informed consent was obtained from all participants prior to questionnaire administration. Participation was entirely voluntary, and confidentiality was upheld throughout data analysis.

### 2.4 Limitations

The main limitation of this study is its cross-sectional design, which captures data at a single point in time. A longitudinal survey would have provided a deeper understanding of the impacts of infectious disease outbreaks over time. Additionally, the findings rely on self-reporting data, which may be influenced by social desirability bias and/or participants' willingness to share information and disclose sensitive information. In the Guinean sociocultural context, norms surrounding gender roles and expectations may have shaped how men and women reported their experiences, potentially leading to the underreporting of certain vulnerabilities, coping strategies, or household dynamics.

The research setting may also have influenced participants' responses. Data were collected within community environments where social hierarchies, trust in researchers, and the timing of the study relative to recent outbreak experiences could have affected the openness with which impacts were discussed. Participants may have emphasized socially acceptable narratives or minimized experiences perceived as stigmatizing.

Another limitation is the gender imbalance of the sample, which may affect the generalizability of certain findings, particularly those concerning men. Despite these limitations, the study makes an important contribution by capturing both qualitative and quantitative perspectives on a topic that remains under-researched in Guinea. Future studies could mitigate potential reporting biases by triangulating self-reported data with longitudinal follow-up, ethnographic observation, and administrative or community-level records, as well as by incorporating gender-sensitive and culturally adapted data collection strategies.

## 3 Findings

The study sample consisted mainly of young adults, with a median age of 30 years, and a predominance of women. A detailed overview is presented in Table 2.

**Table 2:** Sociodemographic Characteristics of the Study Sample.

	Overall (N = 906)	Stratified by Gender		
Characteristic	*N* = 906^1^	Female, *n* = 649^1^	Male, *n* = 257^1^	*p*-value^2^
**Age (median, IQR)**	30.0 (22.0, 41.8)	30.0 (22.0, 43.0)	28.0 (22.0, 40.0)	0.251
**Age group**				0.012
18–29 years	433 (47.8%)	304 (70.2%)	129 (29.8%)	
30–49 years	302 (33.3%)	207 (68.5%)	95 (31.5%)	
50 years and older	171 (18.9%)	138 (80.7%)	33 (19.3%)	
**Gender**				
Female	649 (71.6%)			
Male	257 (28.4%)			
**Education level**				<0.001
No education	284 (31.3%)	246 (86.6%)	38 (13.4%)	
Primary	151 (16.7%)	123 (81.5%)	28 (18.5%)	
Vocational/Secondary	357 (39.4%)	227 (63.6%)	130 (36.4%)	
Upper secondary/University	114 (12.6%)	53 (46.5%)	61 (53.5%)	
**Profession**				<0.001
Civil servant	108 (11.9%)	66 (61.1%)	42 (38.9%)	
Agriculture/Forestry/Fishing	20 (2.2%)	12 (60.0%)	8 (40.0%)	
Commerce/Business	176 (19.4%)	153 (86.9%)	23 (13.1%)	
Unemployed/Housewife	209 (23.1%)	170 (81.3%)	39 (18.7%)	
Artisan/Worker	216 (23.8%)	141 (65.3%)	75 (34.7%)	
Other	177 (19.5%)	107 (60.5%)	70 (39.5%)	
**Ethnicity**				0.144
Forest ethnicity (indigenous)	105 (11.6%)	74 (70.5%)	31 (29.5%)	
Malinké	229 (25.3%)	172 (75.1%)	57 (24.9%)	
Peulh	271 (29.9%)	203 (74.9%)	68 (25.1%)	
Soussou	230 (25.4%)	153 (66.5%)	77 (33.5%)	
Other	71 (7.8%)	47 (66.2%)	24 (33.8%)	
**Religion**				0.030
Other	19 (2.1%)	16 (84.2%)	3 (15.8%)	
Catholic	40 (4.4%)	22 (55.0%)	18 (45.0%)	
Muslim	847 (93.5%)	611 (72.1%)	236 (27.9%)	

^1^ IQR: interquartile range.

^2^Wilcoxon-Mann-Whitney test; Chi-square test of independence.

One qualitative question (Q36) was administered to understand the impacts of infectious diseases outbreaks on households. Table 3 presents the sociodemographic characteristics of the 508 respondents to that question.

**Table 3:** Sociodemographic characteristics of Q36 qualitative question of the survey.

Profile of Q36 respondents (qualitative explanation of impacts)	*N* = 508
**Age (median, IQR)**	30.0 (23.0–41.0)
**Age group**	
18–29 years	236 (46.5%)
30–49 years	181 (35.6%)
50 years and older	91 (17.9%)
**Gender**	
Female	366 (72.0%)
Male	142 (28.0%)
**Education level**	
No education	159 (31.3%)
Primary	75 (14.8%)
Vocational/Secondary	203 (40.0%)
Upper secondary/University	71 (14.0%)
**Profession**	
Civil servant	71 (14.0%)
Agriculture/Forestry/Fishing	13 (2.6%)
Commerce/Business	111 (21.9%)
Unemployed/Housewife	105 (20.7%)
Artisan/Worker	111 (21.9%)
Other	97 (19.1%)
**Ethnicity**	
Forest ethnicity (indigenous)	67 (13.2%)
Malinké	161 (31.7%)
Peulh	113 (22.2%)
Soussou	127 (25.0%)
Other	40 (7.9%)
**Religion**	
Other	11 (2.2%)
Catholic	26 (5.1%)
Muslim	471 (92.7%)

^1^IQR: interquartile range.

To better assess the impacts of infectious disease outbreaks on households, a question was included to explore the nature and distribution of family responsibilities among respondents. Table 4 shows that family responsibilities such as meal preparation, childcare, and caring for the sick are predominantly assumed by women, with statistically significant gender differences, while tasks like animal care and other responsibilities show no significant gender disparity.

**Table 4:** Distribution of family responsibilities.

	Overall	Stratified by Gender		
Family responsibilities	N = 906	Female, *N* = 649	Male, *N* = 257	*p*-value
**Animal care**				0.577
No	890 (98.2%)	636 (71.5%)	254 (28.5%)	
Yes	16 (1.8%)	13 (81.3%)	3 (18.8%)	
**Meal preparation**				<0.001
No	605 (66.8%)	355 (58.7%)	250 (41.3%)	
Yes	301 (33.2%)	294 (97.7%)	7 (2.3%)	
**Childcare**				<0.001
No	639 (70.5%)	399 (62.4%)	240 (37.6%)	
Yes	267 (29.5%)	250 (93.6%)	17 (6.4%)	
**Care for the sick**				<0.001
No	782 (86.3%)	534 (68.3%)	248 (31.7%)	
Yes	124 (13.7%)	115 (92.7%)	9 (7.3%)	
**Other responsibility**				0.558
No	856 (94.5%)	615 (71.8%)	241 (28.2%)	
Yes	50 (5.5%)	34 (68.0%)	16 (32.0%)	
**None of these responsibilities**				<0.001
No	384 (42.4%)	342 (89.1%)	42 (10.9%)	
Yes	522 (57.6%)	307 (58.8%)	215 (41.2%)	

The reported distributions reflect primary responsibility rather than participation in tasks. In many households, domestic and caregiving duties are shared among multiple members, and respondents may have answered “No” when they were not the main person responsible, even if they contributed regularly.

### 3.1 Government measures and impacts on households

One of the survey questions focused on participant's perceptions of government measures implemented during infectious disease outbreaks such as COVID-19, Ebola, and others. Overall, most respondents rated government measures as excellent (44,7%) or adequate (40,7%). Men were more likely than women to rate the measures as excellent (51.4% vs. 42.1%), while women more often rated them as adequate (43.0% vs. 35.0%). Negative ratings and uncertainty were low and similar across genders, with differences not reaching statistical significance (*p* = 0.061).

Overall, 41.6% reported being affected, mainly younger, more educated individuals and those from minority groups. Gender and occupation were not associated with the impacts. Table 5 summarizes the distribution of responses by gender and other sociodemographic characteristics.

**Table 5:** Characteristics of individuals affected by infectious disease outbreaks, and the measures implemented by the government.

Characteristics	Not affected (*n* = 529)	Affected (*n* = 377)	p-value
Age (median, IQR)	30.0 (21.0, 45.0)	30.0 (23.0, 40.0)	0.976
Age group			<0.001
18–29 years	257 (59.4%)	176 (40.6%)	
30–49 years	153 (50.7%)	149 (49.3%)	
50 years and older	119 (69.6%)	52 (30.4%)	
Gender			0.071
Female	391 (60.2%)	258 (39.8%)	
Male	138 (53.7%)	119 (46.3%)	
Education level			0.001
No education	183 (64.4%)	101 (35.6%)	
Primary	100 (66.2%)	51 (33.8%)	
Vocational/Secondary	189 (52.9%)	168 (47.1%)	
Upper secondary/University	57 (50.0%)	57 (50.0%)	
Profession			0.178
Civil servant	54 (50.0%)	54 (50.0%)	
Agriculture/Forestry/Fishing	11 (55.0%)	9 (45.0%)	
Commerce/Business	105 (59.7%)	71 (40.3%)	
Unemployed/Housewife	136 (65.1%)	73 (34.9%)	
Artisan/Worker	123 (56.9%)	93 (43.1%)	
Other	100 (56.5%)	77 (43.5%)	
Ethnicity			0.003
Forest ethnicity (indigenous)	59 (56.2%)	46 (43.8%)	
Malinké	150 (65.5%)	79 (34.5%)	
Peulh	163 (60.1%)	108 (39.9%)	
Soussou	129 (56.1%)	101 (43.9%)	
Other	28 (39.4%)	43 (60.6%)	
Religion			0.788
Other	10 (52.6%)	9 (47.4%)	
Catholic	22 (55.0%)	18 (45.0%)	
Muslim	497 (58.7%)	350 (41.3%)	

To delve deeper into the core theme of this study, respondents who reported being affected by government measures were asked about the types of impacts they experienced. Table 6 presents the reported impacts by gender. Psychological, economic, and cultural impacts showed statistically significant gender differences, with women more frequently reporting psychological impacts, while men were more likely to report economic and cultural impacts.

**Table 6:** Type of impacts by gender.

Type of impacts	Female, *N* = 258	Male, *N* = 119	p-value
**Psychological Impact**			0.002
No	191 (64.5%)	105 (35.5%)	
Yes	67 (82.7%)	14 (17.3%)	
**Social Impact**			0.359
No	70 (72.2%)	27 (27.8%)	
Yes	188 (67.1%)	92 (32.9%)	
**Economic Impact**			0.010
No	160 (73.7%)	57 (26.3%)	
Yes	98 (61.3%)	62 (38.8%)	
**Cultural Impact**			0.020
No	206 (71.5%)	82 (28.5%)	
Yes	52 (58.4%)	37 (41.6%)	
**Other**			0.631
No	237 (68.1%)	111 (31.9%)	
Yes	21 (72.4%)	8 (27.6%)	

Other impacts mentioned by participants included physical health impacts (e.g., discomfort wearing a facial mask) or specific impacts related to work, education, transport and security.

### 3.2 Lived experiences of sociocultural and economic disruption during health crises

A qualitative question was posed following the previous quantitative items to further explore the nature of the impacts experienced. The thematic analysis identified several key themes, including no impact, isolation and stigmatization with loss of social support, mental health effects, fear, economic precarity and monetary borrowing, food insecurity, cultural changes such as altered funeral rites, increased care work, violence, and school closures. For this article, we focus specifically on the socioeconomic impacts. The qualitative analysis presents the most common themes emerging from participants' testimonies, focusing on two main areas: the impacts on domestic and care responsibilities, and the pressure faced by household economies.

#### 3.2.1 Impacts on domestic and care responsibilities

Some participants reported no significant impacts on their lives. For example, a woman explained, “Everything was as before, nothing changed except that we stayed without working, but we managed” (Woman #290). Another respondent, a woman, described not only her personal resilience but also the structural reality of women's everyday lives, where heavy caregiving and household responsibilities are constant, not just crisis-driven:

“As a woman, I can't say that the disease had an impact on my activities, because I've been used to this since childhood. And as a woman, you have to work to be able to handle everything, and it all depends on proper planning. I wake up at 5 a.m. to cook, and by 7 a.m. I'm done. Then I get the children ready for school, and at 2 p.m. we're back home, and study time starts at 6 p.m. They go to bed at 8 p.m. So, this situation couldn't stop me from working” (Woman #320).

Traditional gender roles in household tasks remained firmly in place during the COVID-19 pandemic, with many women emphasizing that these responsibilities were simply a continuation of what they had always done. As one woman explained: “It's very simple: because we are women, cleanliness is our responsibility” (Woman #189).

The findings do not explicitly indicate a greater family caregiving burden among men. The few responses referring to men emphasized their role in restricting children's movements due to infection risks and supervising them (n = 5), helping review school lessons (n = 1), and assisting with meal preparation or market errands to support mothers, particularly among adult sons still living at home (n = 2).

On the other hand, the most frequently reported impact in the qualitative analysis was an increased burden of care work, particularly among women. Many respondents described how COVID-19 pandemic-related disruptions, such as school closures and increased household responsibilities, intensified their caregiving roles. For instance, a university student recounted how she had to take on full-time caregiving duties for her sister's children while continuing her studies remotely:

“At that time, I was taking care of my sister's children and managing things at home because she's a doctor and was going to the hospital, so she wasn't home much. I was a university student, and the universities were closed, and we were doing online classes. That's what increased my responsibilities. Everything she usually did for her children during the day; I was the one doing it. I could only get some rest when she came back at night” (Woman #108).

This increased care work was not limited to women. Some male respondents also described stepping into caregiving roles, particularly for younger siblings. One man shared: “I prepared the meals when my little sisters went to school and when our mother was either out or not feeling well. I would go to the market to buy the ingredients, then come back to cook. If something was missing, the neighbors would try to help me out” (Man #024).

Many other testimonies also highlighted the additional burden of care work for siblings during the COVID-19 pandemic. As another respondent explained: “I would go to school, and when I came back, I looked after my little brothers. I told them to keep their distance from their classmates and not to put their hands in their mouths” (Woman #912). Children also took on more domestic chores to support their families during long periods at home. As one woman noted: “Since the children were at home all the time, they helped me with the household chores” (Woman #296).

Because of economic uncertainty, some women were forced to adjust their daily routines and spend longer hours at the market to try to sell more. This often shifted household responsibilities to older children. As one woman recounted: “When my children came home from school, the eldest would cook. I wasn't stable at that time. When I left for the market, I only came back in the evening, so my daughter prepared meals for her brothers” (Woman #092).

Among polygamous couples, some women reported that the burden of household and caregiving duties was shared with co-wives, allowing for a rotation of responsibilities. One woman explained: “I cooked, and it was done in turns with my co-wife, and I also had my small business. I looked after the children as well” (Woman #646). Similarly, another respondent noted: “The cooking was done in turns. There are three of us wives with our husband” (Woman #592).

Some women adjusted their care work depending on the health and availability of co-wives. As one described: “At that time, I wasn't working. My co-wife was unwell and had foot pain, so I took care of her. I was at home all the time” (Woman #145). Others highlighted structured rotations for household duties to ensure everyone contributed: “If you stay at home, it's difficult because you won't have anything. Here, each of us prepares meals two days a week; there are three of us wives” (Woman #155).

Finally, one respondent emphasized how shared duties extended beyond cooking to childcare and protection from disease: ““Since there are three of us, cooking was done in turns. I wasn't the only one cooking. I took care of the children to protect them from this disease, and I did the same for the elderly” (Woman #749).

These testimonies indicate that, in polygamous households, care work and domestic responsibilities could be distributed among co-wives, helping to manage the combined demands of childcare, eldercare, and income-generating activities during the COVID-19 pandemic.

Household economies under pressure.

Economic hardship emerged as an important theme in the qualitative analysis. Many participants described how the infectious disease outbreaks severely disrupted livelihoods, increased debts, and compromised food security within their households.

For instance, a woman recounted how difficult it became to secure enough food for her and her family: “During Corona, it was so difficult that we only prepared cassava to eat at home. I wasn't selling anything; it was only when I went to the forest and came back that I would buy cassava to cook at home. We didn't cook rice all the time because it was too hard” (Woman #059).

Similarly, another woman reflected on the daily struggle to find enough food to eat and the reliance on divine providence:

“Sometimes we have something to eat, and sometimes we don't. In the morning, we don't go out, so we see if we can find something to eat with the little, we have at home, and if we don't have anything, we just don't eat, because no one dies unless God wills it—hunger doesn't kill. I go to work, and my income depends on God's grace” (Woman #177).

For many women, however, survival during the crisis depended not only on faith but also on financial support from their husbands, reflecting a loss of economic independence: “It wore me out—I wasn't earning any money. If my husband took pity on me, he gave me a little, but I wasn't earning anything myself, and it destroyed all my savings.” (Woman #385).

The COVID-19 pandemic disrupted daily livelihoods, particularly for women engaged in informal trade. One woman explained:

“When we went to the market, there weren't enough goods available. Even if you didn't want to go out, you had no choice; otherwise, you couldn't eat, and the children stayed home. I had a younger sister who looked after them. I'm a vendor and usually spend half my day at the market, but with this disease there were fewer buyers, and I had to return home quickly” (Woman #446).

This economic strain was echoed by others who saw their savings evaporate. As another woman recounted: “During Ebola, we suffered a lot. All our savings were gone because, imagine, the mosques were closed, people could not gather, and as vendors, we rely on crowds for business. Every seller had to leave by 8 p.m., but I used to sell at night, so this reduced my income” (Woman #970). In some cases, women's ability to earn an income was further limited by household power dynamics. As one woman explained:

“It was difficult because my husband refused to let me run my small business. He forbade me from doing any activity—even sewing; he wouldn't allow me to go to the workshop. He gave me money for household expenses, and we managed with that. I was doing everything at home, and sometimes my mother helped me when the children came back from school” (Woman #357).

These accounts illustrate how market restrictions, income loss, and even intra-household constraints combined to undermine women's economic autonomy during the COVID-19 pandemic, while leaving them responsible for both childcare and domestic work.

Economic precarity often translated into significant food insecurity and accumulation of debt. One participant described how she tried to manage household needs by borrowing and reselling food, which ultimately led to financial distress:

“We were selling rice and getting into debt with people at the market to feed our children. When we bought the rice, we would grind it into flour and resell it, hoping to make a profit. I ended up owing more than 6 millions of our francs, and the owners keep asking me to pay them back. To be able to treat my children when they were sick, I spent two years hiding from those who had lent me money. I have more than 12 children. My husband was a driver, but he had an eye operation, and since then, I've been the one taking care of the family. That's why I've faced all these difficulties. When I go to bed, I can't sleep because I keep thinking, ‘How will we eat tomorrow?’ Otherwise, I used to be fat, but now I am thin” (Woman #930).

In some cases, these economic pressures led to informal monetary borrowing from neighbors and relatives to cover essential health-related expenses. As one woman explained:

“We kept a close watch on the children to protect them from the disease because the time we were living in was not good. And when a child got sick, if we didn't have money, we would borrow from a neighbor to take them to the hospital, and when we earned some money, we would pay it back. Because if your child falls ill, you also fall ill psychologically” (Woman #955).

#### 3.2.2 Coping mechanisms amid care and financial burdens

Participants were asked about the consequences of infectious disease outbreaks on their households, specifically in terms of coping behaviors adopted during the crisis. Table 7 shows the frequency of reported behaviors, regardless of gender, as respondents were speaking about households' strategies rather than personal ones.

**Table 7:** Coping behaviors adopted by households during infectious disease outbreaks.

Behaviors adopted during infectious disease outbreaks	N = 906
**Reduce food consumption**	
No	553 (61%)
Yes	353 (39%)
**Reduce food quality**	
No	626 (69%)
Yes	280 (31%)
**Send children to live elsewhere**	
No	878 (97%)
Yes	28 (3.1%)
**Marry off household girls**	
No	890 (98%)
Yes	16 (1.8%)
**Seek free food/beg**	
No	863 (95%)
Yes	43 (4.7%)
**Receive government aid**	
No	835 (92%)
Yes	71 (7.8%)
**Take on additional work**	
No	875 (97%)
Yes	31 (3.4%)
**Send children to work/beg**	
No	898 (99%)
Yes	8 (0.9%)
**Borrow (family/friends)**	
No	815 (90%)
Yes	91 (10%)
**Borrow (institutions)**	
No	887 (98%)
Yes	19 (2.1%)
**Pawn assets**	
No	895 (99%)
Yes	11 (1.2%)
**Sell or rent assets**	
No	897 (99%)
Yes	9 (1.0%)
**Not applicable**	
No	479 (53%)
Yes	427 (47%)

^1^n (%).

These findings reveal that infectious disease outbreaks in Guinea have multifaceted impacts on households, such as increased care burdens and economic precarity. These results raise important questions about the broader social consequences of epidemics and strategies to cope.

## 4 Discussion

This study shed light on the multidimensional impacts of infectious disease outbreaks on the Guinean population and how these were experienced and perceived by gender. The research has demonstrated that gender plays a role in molding individuals' roles and responsibilities within communities and households. These gender-related dynamics have the potential to impact one's access to resources, healthcare services, and decision-making authority.

From an intersectional lens, the findings highlight the heightened vulnerability observed among adults aged 30–49 years. This may reflect their dual economic and caregiving responsibilities, making them particularly sensitive to disruptions caused by public health measures, especially the ones during the COVID-19 pandemic. Similarly, individuals with vocational or secondary education may occupy occupations with less flexibility or job security, amplifying their exposure to economic and social stressors. In the Guinean context, family roles and expectations are shaped by the intersection of traditions, sociocultural norms, gender relations, and socioeconomic conditions. Vulnerabilities are therefore mediated not by gender alone but by how gender interacts with religious interpretations, education levels, and livelihood realities to influence caregiving duties, mobility, and health-seeking behaviors. For example, domestic tasks are often framed as women's responsibilities, and men's participation may be socially discouraged; however, practices vary across region, religious interpretation, and women's education level [26]. These intersecting factors shape access to resources and decision-making power and should be considered in infectious disease preparedness strategies, which must address overlapping vulnerabilities related to gender, age, education, and occupation rather than relying solely on gender-based assumptions.

A key finding of our study, highlighted in Table 6, reveals a potential inconsistency between quantitative and qualitative data. Quantitatively, men reported significantly higher levels of economic impact (*p* = 0.010). However, the qualitative findings are dominated by detailed testimonies from women describing severe financial distress that led to food insecurity and mental health distress. This apparent contradiction suggests that men and women may differ in how they perceive, define, or report socioeconomic impacts. Religious and sociocultural expectations surrounding masculinity, patriarchy, family provision, and female modesty may also shape how hardship is articulated or minimized in public discourse. While survey data capture self-reported impacts in a standardized format, qualitative narratives allow for a richer exploration of lived experiences. This divergence underscores the importance of integrating mixed methods. It invites a deeper analysis and discussion on gendered perceptions of economic hardship and the possible influence of social norms, reporting bias, or contextual factors on how impacts are expressed.

### 4.1 Increased burden of care work

Both quantitative and qualitative data point to an increased burden of care work disproportionately affecting women, which aligns with the broader trend observed in our sample, where caregiving responsibilities were primarily undertaken by women. For example, the COVID-19 pandemic intensified care responsibilities, with many respondents taking on full-time caregiving for children, siblings or other family members while managing school or work. In polygamous households, shared duties among co-wives helped distribute the burden. These findings highlight how household composition and intra-family support shaped the management of care work during the crisis. Such arrangements are embedded in social and religious norms governing marriage, kinship, and mutual support, which may both redistribute care responsibilities and reinforce expectations that women assume primary caregiving roles [27], [28].

Importantly, qualitative testimonies suggest that women's heavy domestic and caregiving workload is often normalized as part of everyday life rather than perceived as an additional burden during crises [27]. Because these responsibilities are deeply embedded in gendered social expectations, women may not identify an increased workload during epidemics, even when demands intensify. This normalization helps explain why some participants reported little change in their daily responsibilities despite heightened care demands. Moreover, when describing sources of support, respondents referred primarily to other women, including sisters, co-wives, daughters, and older children, rather than husbands, indicating that caregiving responsibilities remain largely structured within female solidarity networks and reflecting the limited involvement of male partners in routine domestic support.

Gender roles shape the division of labor within households and communities, influencing responsibilities such as income generation, food preparation, childcare, animal care, and water collection [7], [29]. They also define socially constructed rights, obligations, and expectations, as well as culturally embedded beliefs about appropriate behaviors for men and women [29]. These norms influence health-related decision-making, including when and how individuals seek care. In Guinea, these decisions are further shaped by the coexistence of tradition and modernity, negotiated differently across social groups: urban elites and educated populations tend to rely on modern medicine/family planning and formal health services, while poorer households face economic barriers alongside a growing demand for quality care [30]. Persistent gender inequalities further constrain access and decision-making power, as women's labor force participation remains lower than men's (41.6% vs. 63%), nearly half marry before age 18, only about one-third participate in major household decisions, and literacy rates remain substantially lower among women [31].

Gendered divisions of labor at the human–animal interface are documented in West Africa [32], with men typically responsible for large livestock and women for small animals; however, these patterns did not emerge prominently in this study, likely reflecting the predominantly urban and peri-urban settings of participating households. Complementary research within the same research project (DOPERAUS) explored gendered animal-related practices more explicitly through focus groups with hunters and women involved in bushmeat vending, underscoring the importance of disaggregating responsibilities by species and gender to better understand exposure pathways and OH risks.

A connection can be established between caregiving and the resilience of communities, as women frequently assume a central role in caregiving both within families and broader communities. Their responsibilities include caring for children, the elderly, and the ill, which can impact their susceptibility to pathogens and their capacity to effectively respond to infectious disease outbreaks. Some research has already explored gendered social interactions, focusing on the roles and cultural expectations associated with each sex/gender [33], [34]. Traditionally, women have been expected to fulfill caregiving roles, not only in their family responsibilities but also as the primary caregivers during times of illness [35], as shown in the post-Ebola context in Guinea [36]. Acknowledging the caregiving roles of women is a pivotal aspect of enhancing community resilience and readiness. Interactions occurring within both the health system and the community are shaped by constraining gender norms and disparities, including issues related to power dynamics and trust [37]. As mentioned by the authors, these factors have an impact on the effectiveness, efficiency, and overall performance. Faith leaders and religious networks can also influence caregiving practices and prevention behaviors by legitimizing health measures and reinforcing collective responsibility.

The findings highlight the gendered nature of the socioeconomic impacts of epidemics, showing that women and men experience these effects differently. Women were more likely to report impacts related to caregiving responsibilities, disruptions to household management, and reduced access to resources and markets, reflecting their central role in domestic and care activities, and the impacts on their income-generating activities. Men, in contrast, more frequently reported economic and cultural impacts, including loss of income and disruptions to traditional leadership roles. These patterns align with prevailing gender norms in Guinea [27], [28], [38], where men are primarily responsible for household financial provision and women for domestic responsibilities, including caregiving. Infectious disease outbreaks disrupt daily routines, work patterns, and economic life, slowing everyday activities and making inequalities more visible, particularly within households where people spend most of their time [18]. Shaped by social norms as well as religious and community structures that influence authority, decision-making, and access to resources [27], these gendered expectations underscore how epidemics exacerbate existing socioeconomic inequalities across genders.

### 4.2 Economic impacts and precarity

The findings echo existing literature, demonstrating that the economic impacts of health crises like Ebola and COVID-19 have disproportionately affected women [17], [39]. Our results show that women in Guinea more frequently reported economic hardships, which aligns with broader patterns where women, particularly those heading low-income or single-parent households, are more likely to work in precarious or informal jobs with limited protections [40]. The school and nursery closures also impact women's participation in the paid economy [40]. These jobs were among the first to be disrupted during the COVID-19 pandemic, directly contributing to reduced income, food insecurity, and financial stress, as reflected in both the qualitative and quantitative data. Lower levels of formal education among women [31] in some regions further constrain access to stable employment and health information, reinforcing structural inequalities.

Women's overrepresentation in the care sector and caregiving roles also exposed them to greater health and financial risks during outbreaks [15], [41]. At the same time, gendered disparities in socioeconomic status constrained women's ability to access healthcare and other social protections, further compounding their vulnerability. Conversely, higher educational attainment appears to enhance women's autonomy, health literacy, and capacity to navigate prevention measures and healthcare services, suggesting a protective effect that interacts with cultural and religious expectations.

One critical coping strategy reported by participants was borrowing money, formally or informally, to meet basic needs such as food, healthcare, or children's education. Some women described relying on neighbors or community members for short-term loans, often without interest or collateral, while others accumulated significant formal debts, sometimes leading to long-term financial strain, and, in some cases, social stigma. This informal borrowing network served as a key safety net in the absence of institutional support, highlighting the structural limitations of financial protection systems during infectious disease outbreaks in Guinea. These findings suggest that strengthening community-level safety nets and integrating gender-responsive economic policies are essential for increasing household resilience and equity in the face of future health crises.

These findings underscore how gendered economic inequalities not only exacerbate individual hardships but also hinder broader public health efforts. Limited healthcare access and economic insecurity can delay care-seeking behavior and reduce communities' ability to participate in outbreak detection and control. The COVID-19 pandemic showed that the consequences of overlooking gender and its intersecting factors as key determinants of health and well-being are significant. In responding to a crisis, stakeholders often focus on controlling the virus, overlooking the social inequalities that the COVID-19 pandemic has exacerbated [18]. Integrating gender-sensitive economic and health policies into infectious disease outbreaks preparedness and response strategies is therefore critical for improving resilience and equity in future responses [11]. Designing inclusive interventions requires engaging religious leaders, supporting girls' and women's education, and tailoring communication strategies to culturally grounded norms in order to improve prevention and equitable access to care. Strengthening women's education and decision-making power, protecting livestock-based livelihoods, and implementing integrated human–animal surveillance systems can simultaneously reduce zoonotic risks, support household resilience, and advance OH outcomes [9], [11].

Zoonotic diseases often arise at the interface of human, animal, and environmental systems, and OH must be conceived as a systemic, integrative approach that draws on diverse disciplinary insights to generate contextually appropriate interventions [43]. Despite its potential, gender remains a neglected dimension within the OH discourse and practice, and fully engaging women as agents of change is critical [42]. A OH approach that recognizes the intersection of gender, religion, and education can strengthen trust, enhance community engagement, and ensure that prevention and response strategies are socially responsive and equitable. These findings demonstrate that addressing gendered socioeconomic vulnerabilities is a key component of resilient and effective OH strategies in infectious disease outbreaks preparedness and response.

Beyond documenting disparities, these findings highlight the critical role of social sciences in advancing more equitable and effective OH strategies [29], [44], [45]. Social science insights can inform community-led actions such as shared caregiving models, women's support networks, and economic empowerment initiatives that strengthen household resilience and reduce gendered vulnerabilities. Participatory approaches can also help identify culturally acceptable ways to engage men in caregiving and health decision-making. Participatory, field-based approaches can strengthen OH strategies by helping collaboration among sectors and disciplines, and engaging communities and frontline actors in identifying locally acceptable interventions [46]. Positioned as instruments of change rather than solely diagnostic tools, social sciences can help reframe OH practice by addressing the social norms, power dynamics, and structural inequities that shape exposure, prevention, and care [47]. A socially responsive OH approach should therefore ensure equitable access to prevention, control, and resilience-building efforts through meaningful community engagement.

## 5 Conclusion

This study demonstrates the gendered socioeconomic impacts of infectious disease outbreaks in Guinea, with women disproportionately affected by caregiving responsibilities and economic precarity, and men more impacted by income loss and disruptions to traditional gender roles as financial providers. Vulnerabilities were shaped not only by gender but also by age, occupation, and socioeconomic position.

These findings underscore the importance of incorporating social science perspectives into the OH approach. Understanding household dynamics, community roles, local belief systems, and economic inequalities is essential for designing effective, equitable interventions. In line with recent OH High-Level Expert Panel recommendations, anthropological and participatory research can help identify risk behaviors, assess the acceptability and feasibility of mitigation measures, and integrate gender considerations while drawing on local and Indigenous knowledge systems [45]. Public health and OH researchers must therefore embrace intersectional approaches that consider overlapping identities, structural inequities, and historically marginalized groups [48]. Valuing local knowledge and community practices, alongside scientific evidence, can strengthen trust, improve intervention uptake, and ensure culturally appropriate responses [44]. Integrating gender-sensitive policies and strengthening community-level safety nets can enhance both population resilience and the effectiveness of outbreak prevention and response under a holistic OH framework.

## CRediT authorship contribution statement

**Stéphanie Maltais:** Writing – review & editing, Writing – original draft, Visualization, Validation, Supervision, Project administration, Methodology, Funding acquisition, Formal analysis, Data curation, Conceptualization. **Janyck Beaulieu:** Writing – original draft, Validation, Formal analysis. **Castro Gbêmêmali Hounmenou:** Writing – review & editing, Validation, Methodology, Investigation, Formal analysis, Data curation. **Salifou Talassone Bangoura:** Writing – review & editing, Validation, Investigation, Formal analysis. **Gnouma Laurent Koniono:** Writing – review & editing, Validation. **Emile Faya Bongono:** Writing – review & editing, Validation, Formal analysis. **Alioune Camara:** Writing – review & editing, Validation, Supervision, Project administration, Funding acquisition. **Justin Masumu:** Writing – review & editing, Project administration, Funding acquisition, Conceptualization. **Abdoulaye Touré:** Writing – review & editing, Validation, Supervision, Project administration, Funding acquisition.

## Ethics declaration

### Informed consent and patient details

Written informed consent to take part in the study and to publish the article has been obtained from all participants or their legal representatives. The privacy rights of participants have been observed.

### Studies in Human

This study was performed in compliance with relevant laws, regulatory frameworks and guidelines where the research took place.

This study was approved by the The Guinean National Health Research Ethics Committee approved this study (authorization no. 025/CNERS/23). Ethical approval was granted by the University of Ottawa (S-10-22-8549), where one of the authors was formerly affiliated, and this approval was later acknowledged by the Research Ethics Committee in Science and Health (CERSES) at the Université de Montréal (Project 2026–8316).

(Approvals No. 025/CNERS/23; S-10-22-8549; 2026–8316).

## Funding

This study was supported by Canada's International Development Research Centre (IDRC), Canada (Grants No. 109812 and No.109976-006).

## Declaration of competing interest

The authors declare that they have no known competing financial interests or personal relationships that could have appeared to influence the work reported in this paper.

## Data Availability

The de-identified dataset contains potentially sensitive information and is not publicly available. Access to the data may be granted on a case-by-case basis, subject to formal request and in compliance with the ethical approvals obtained from the Guinean National Health Research Ethics Committee, the University of Ottawa, and the Université de Montréal. Requests must be justified, reviewed, and approved in accordance with these ethical frameworks, and should be addressed to the corresponding author.
